# Overview of Gene Special Issue “Genetic Conditions Affecting the Skeleton: Congenital, Idiopathic Scoliosis and Arthrogryposis”

**DOI:** 10.3390/genes13071194

**Published:** 2022-07-04

**Authors:** Philip F. Giampietro, Nancy Hadley-Miller, Cathy L. Raggio

**Affiliations:** 1 Department of Pediatrics, University of Illinois-Chicago, Chicago, IL 60612, USA; 2 Musculoskeletal Research Center, Children’s Hospital Colorado, Aurora, CO 80045, USA; nancy.miller@childrenscolorado.org; 3 Hospital for Special Surgery, New York, NY 10021, USA; raggioc@hss.edu

## Introduction

In this Special Issue of *Genes* entitled “Genetic Conditions Affecting the Skeleton: Congenital, Idiopathic Scoliosis and Arthrogryposis”, evidence is presented which suggests that congenital, idiopathic scoliosis, and arthrogryposis share similar overlapping, but also distinct etiopathogenic mechanisms, including connective tissue and neuromuscular mechanisms. Congenital scoliosis (CS) is defined by the presence of an abnormal spinal curvature due to an underlying vertebral bony malformation (VM). Idiopathic scoliosis (IS) is defined by the presence of an abnormal structural spinal curvature of ≥10 degrees in the sagittal plane in the absence of an underlying VM. Arthrogryposis is defined by the presence of congenital contractures in two or more joints of the appendicular skeleton. All three conditions have complex genetic causes. This Special Issue highlights the complex nature of these conditions and current concepts in our approach to better understand their genetics.

In 2007, Yves Cotrel postulated genetic, biomechanic, neurological, oto-rhino-laryngology, molecular biology, endocrinology, neurophysiology, biochemistry, sensory physiology, and anatomical pathological mechanisms for the occurrence of IS [1]. GWAS studies have confirmed the complexity of IS and the existence of low-penetrance genes associated with IS. Genetic loci in muscle developmental genes (*LBX1* and *BNC2*) and extracellular matrix genes (*FBN1*) have been identified as potential predisposition genes for IS. Additional evidence for cilia-related genes in the development of AIS has been presented in this Special Issue by Mathieu et al. [2] with autosomal dominant transmitted *POC5* coding gene variants occurring at a higher frequency in patients with AIS as compared with the general population. *POC5* plays an important role in centriole assembly.

Terhune et al. [3] hypothesize that the development of IS may be due to damaging variants within a specific set of pathways or molecular classes, rather than being driven by just a few select ‘AIS genes’. Performing whole-exome sequence analysis on 23 multi-generation families, the authors identified an enrichment of variants in cytoskeletal- and extracellular-matrix-related processes. One hundred and thirty-two genes were shared by two or more families. Ten genes were shared by >4 families, although no genes were shared by all, supporting the polygenic nature of IS. The combination of the inability to relate specific genetic variants to IS development suggests a potential role of environmental and/or epigenetic factors in the etiology of IS.

Epigenetic causes need to be considered as modulating factors for curve progression in IS. Using discordant monozygotic twin pairs for IS, Carry et al. [4] identified 57 differentially methylated regions (DMRs) where hyper- or hypo-methylation was consistent across the region and 28 DMRs had a consistent association with curve severity. Twenty-one DMRs were correlated with bone methylation, including WNT10A (WNT signaling) and NPY (regulator of bone and energy homeostasis).

In this Special Issue, Janusz et al. [5] studied gender differences associated with curve progression in IS. Genetic differences in *ESR1* and *ESR2* have been hypothesized to be a contributing factor in some cases for AIS. Using muscle tissue obtained from the paravertebral muscles of girls with AIS, differences in DNA methylation between patients with a Cobb angle ≤ 70° and >70° in the T-DMR2 region at the concave side of the curvature were identified, suggesting that *ESR1* and *ESR2* DNA methylation may be associated with AIS severity.

The relationship between CS and IS remains an interesting area for hypothesis generation and future research. One hypothesis is that the two conditions may have similar genetic mechanisms. In zebrafish, the depletion of ptk7 results in both congenital and idiopathic scoliosis. In this edition, Su and colleagues [6] provide evidence that mutations in *PTK7*, which functions in canonical and non-canonical Wnt signaling, are associated with the development of both CS and IS. Preliminary evidence is presented that gradations in expression may correlate with CS and IS, with more severe expression being associated with CS and diminished expression being associated with IS. Apart from the 16p11.2 microdeletion, which contains the *TBX6* gene associated with CS, Lai and colleagues studied copy number variants in a cohort of 67 patients with CS [7]. Some of the CNV contained genes such as *DHX40, NBPF20, RASA2*, and *MYSM1*, which have been found to be associated with syndromes characterized by scoliosis or thought to play a role in bone/spine development. They hypothesize that CNVs in consort with single nucleotide variants, in addition to somatic mutation and environmentally mediated effects, contribute to the occurrence of CS. Wang et al. [8]. identified several hypomorphic sequence variants in *FGFR1,* a transmembrane cytokine receptor involved in gastrulation, organ specification, and the patterning of various tissues in patients with mild spine and heart defects with CS.

The search for genes, pathways, and modifier genes for all three conditions is ongoing and may lead to potential therapies. From a total of 908 genes linked with scoliosis and 444 genes linked with AMC identified in the literature, 227 genes were associated with AMC-SC by Latypova and colleagues [9]. This group of genes is associated with a wide range of cellular functions, including transcription regulation, transmembrane receptor, growth factor, and ion channels. Further research using genomics and animal models to identify prognostic factors and therapeutic targets for AMC needs to be implemented. Dahan et al. [10] described the clinical and genetic heterogeneity in patients with multiple pterygium syndrome and scoliosis, harboring mutations in *CHNRG* and *MYH3*.

Due to the overlap in pathophysiology for arthrogryposis and IS, overlapping treatment mechanisms for both conditions can be conceptualized. *MYH3*-associated distal arthrogryposis in humans may be modeled in smyhc1 zebrafish which develop scoliosis as discussed by Whittle and colleagues [11]. Para-aminoblebbistatin inhibits myosin heavy chain ATPase activity, which chemically relaxes the skeletal muscle and prevents the curved phenotype of treated smyhc1 mutant fish. It is anticipated that our continued developing understanding of the genetic causes of these conditions will facilitate the development of targeted therapeutic interventions.

## Data Availability

Not applicable.
